# On Gall-Stones or Cholelithiasis

**Published:** 1897-06

**Authors:** 


**On Gall-stones or Cholelithiasis.** By EDWARD MANSFIELD BROCKBANK, M.D. Pp. x., 301. London: J. & A. Churchill. 1896.

This volume is the outcome of an investigation on the solvent action of various drugs on gall-stones which was carried out in 1892 at the Owens College, Manchester. It gives a good account of the present condition of our knowledge of a disease which is even yet only imperfectly understood. The author has not discovered any specific remedy, but he has some belief in the practical utility of large doses of olive oil; and he prefers the lighter forms of nitrogenous foods to the carbohydrates, fats, and sweets. He has little faith in cholagogues, but he believes that the last decade has seen a great improvement in the prognosis of many cases which cannot be relieved by medicinal treatment: and he describes the indications for surgical aid in the treatment of calculous affections of the biliary passages.

